# Data driven analysis of low frequency spatio-temporal dynamics in resting state MRI (rsMRI) data

**DOI:** 10.1186/1471-2202-13-S1-P108

**Published:** 2012-07-16

**Authors:** Martha Willis, Lukas Hoffman, Alessio Medda, Shella Keilholz

**Affiliations:** 1 Georgia Tech Research Institute, Atlanta, GA, 30306, USA; 2 Emory University and Georgia Institute of Technology, Atlanta, GA 30306, USA

## 

Resting state MRI (rsMRI), based on fluctuations in blood oxygenation level dependent (BOLD) signals, serves as a powerful tool to map networks of “functional connectivity” in the brain even in the absence of task activation or stimulation. The most popular analysis techniques for resting state networks involve region of interest (ROI) correlations or Independent Component Analysis (ICA) approaches where the networks are assumed to be undirected and static over the course of the several minute long scan. Recent studies by Majeed [4] and Chang [5], show that patterns of connectivity exhibit time-varying properties that change significantly over the course of a single scan. Interactions between different areas of the brain exhibit dynamic properties on the order of tens of seconds [4]. This time scale closely corresponds to the temporal scale observed in cognitive processes suggesting that the dynamics of this “background activity” may influence behavior and/or perception. Characterizing and understanding these dynamics presents unique challenges in terms of signal analysis. We are currently optimizing a completely data driven analysis technique based on wavelet features of BOLD time series data.

**Figure 1:**
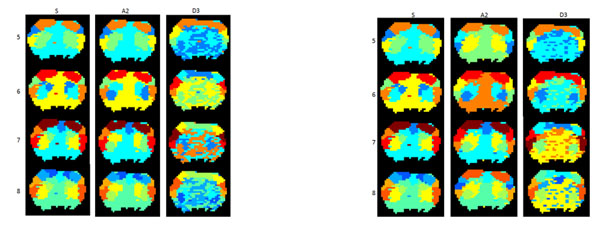
Sample clusters in rat brains. Clusters include original signal (S), A2, and D3 coeffiecients and 5,6,7, and 8 clusters using using two different wavelets. A) Daubechies 12 Wavelet. B) Symlet 8 Wavelet

Wavelets have been used extensively in the analysis of neurological BOLD signals, most commonly, however in task-induced studies with few examples in rsMRI studies. Here we utilize wavelet features of voxel time series, which are clustered using an agglomerative clustering method. The DWT, an algorithm based on subband coding provides a fast computation of the wavelet transform, as in the multiresolution analysis (MRA) algorithm defined by Mallat [6]. A variety of wavelets were compared, and initial results using Daubechies and Symlet wavelets were used to cluster and compare varying numbers of clusters and choice of either approximation (A2) or detail (D3) levels. Figure 1 shows a series of preliminary data for comparison. The successful detection of clusters that match well with classical anatomical boundaries in sensorimotor cortex indicates that wavelet-based segmentation is a promising first step toward data-driven analysis of network dynamics.
